# Mitogen-Activated Protein Kinase Phosphatases (MKPs) in Fungal Signaling: Conservation, Function, and Regulation

**DOI:** 10.3390/ijms20071709

**Published:** 2019-04-05

**Authors:** Gema González-Rubio, Teresa Fernández-Acero, Humberto Martín, María Molina

**Affiliations:** Departamento de Microbiología y Parasitología. Facultad de Farmacia. Instituto Ramón y Cajal de Investigaciones Sanitarias (IRYCIS), Universidad Complutense de Madrid, Plaza de Ramón y Cajal s/n, 28040 Madrid, Spain; gemagonzalezrubio@ucm.es (G.G.-R.); teresafe@farm.ucm.es (T.F.-A.)

**Keywords:** fungal MKPs, MAPKs, signaling, Msg5, Sdp1, Pmp1, Cpp1

## Abstract

Mitogen-activated protein kinases (MAPKs) are key mediators of signaling in fungi, participating in the response to diverse stresses and in developmental processes. Since the precise regulation of MAPKs is fundamental for cell physiology, fungi bear dual specificity phosphatases (DUSPs) that act as MAP kinase phosphatases (MKPs). Whereas fungal MKPs share characteristic domains of this phosphatase subfamily, they also have specific interaction motifs and particular activation mechanisms, which, for example, allow some yeast MKPs, such as *Saccharomyces cerevisiae* Sdp1, to couple oxidative stress with substrate recognition. Model yeasts show that MKPs play a key role in the modulation of MAPK signaling flow. Mutants affected in *S. cerevisiae* Msg5 or in *Schizosaccharomyces pombe* Pmp1 display MAPK hyperactivation and specific phenotypes. MKPs from virulent fungi, such as *Candida albicans* Cpp1, *Fusarium graminearum* Msg5, and *Pyricularia oryzae* Pmp1, are relevant for pathogenicity. Apart from transcriptional regulation, MKPs can be post-transcriptionally regulated by RNA-binding proteins such as Rnc1, which stabilizes the *S. pombe* *PMP1* mRNA. *P. oryzae* Pmp1 activity and *S. cerevisiae* Msg5 stability are regulated by phosphorylation and ubiquitination, respectively. Therefore, fungi offer a platform to gain insight into the regulatory mechanisms that control MKPs.

## 1. Fungi Respond to Distinct Stimuli Through MAPK Pathways

**Figure 1:**
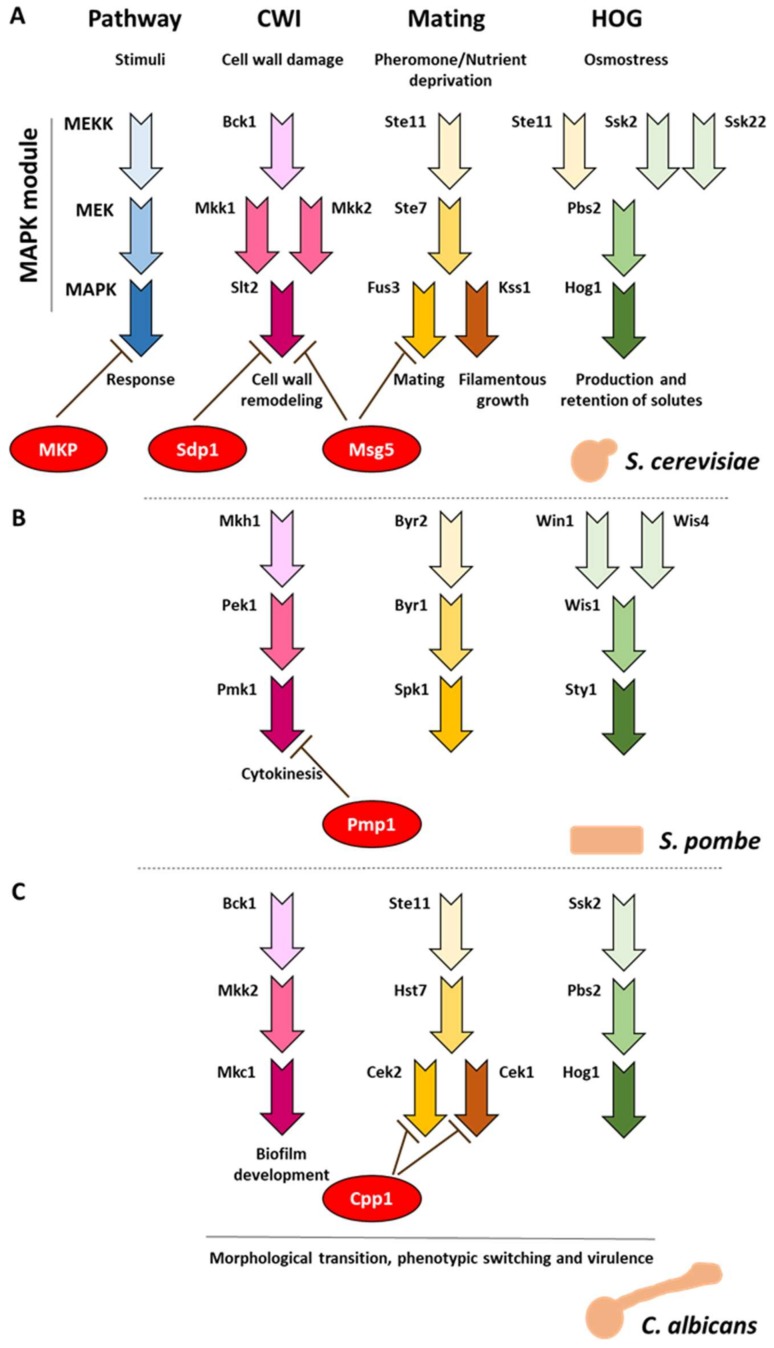
Mitogen-activated protein kinase (MAPK) signaling pathways in model fungi. At the uppermost left side, a schematic view of a MAPK pathway with the components of the MAPK module in blue and the MAP kinase phosphatase (MKP) in red. On the right side (head level), the major MAPK pathways described in fungi, Cell Wall Integrity (CWI), mating/filamentous growth and High Osmolarity Glycerol (HOG), and the stimuli that trigger their activation. The equivalent MAPK pathways are shown for the budding yeast *Saccharomyces cerevisiae* (**A**), the fission yeast *Schizosaccharomyces pombe* (**B**), and the dimorphic yeast *Candida albicans* (**C**). The same code of colors is shown in all cases, as indicated above.

Cells communicate with the environment using an evolved machinery that allows them to interpret an external cue and translate it into an input message that permits the execution of cellular responses. Mitogen-activated protein kinase (MAPK) pathways are one of the main molecular systems involved in this process in eukaryotic organisms. Cells perceive extracellular stimuli, such as hormones, mitogens, and growth factors, through surface receptors attached to the plasma membrane, which transduce the external signal to intracellular proteins. This signal converges in the activation of a MAPK module that is conserved in eukaryotic cells and whose function is the amplification of such signal by sequential events of phosphorylation, making this system sensitive to subtle changes in the cell environment. MAPK pathways regulate a wide variety of cellular processes such as cell growth and division, metabolism, differentiation, and survival [1,2,3] (Figure 1). 

MAPK modules are composed of three protein kinases acting in cascade. At the head level, the serine/threonine (Ser/Thr) kinase MAPKKK (MAP kinase kinase kinase), also known as MAP3K (mitogen-activated protein 3 kinase) or MEKK (MEK kinase), phosphorylates and activates its downstream effector, the MAPKK/MAP2K/MEK. The MEK, in turn, dually phosphorylates both the tyrosine and the threonine residues at the activation loop (Thr-X-Tyr) of the MAPK, which undergoes a conformational change that results in the full activation of the protein [3]. In higher eukaryotes, MAPKs are clustered into five classes: p38, ERK1/2, JNK, ERK5, and atypical MAPKs. The activated MAPK is the final component of the cascade and phosphorylates its substrates in a serine or threonine residue followed by a proline (Ser/Thr-Pro). Many of the MAPK substrates are transcription factors which, upon phosphorylation, adjust the transcriptional pattern of the cell to the particular condition determined by the stimulus. The activity of the MAPK is precisely regulated in the cell, and inappropriate modulation of these pathways is linked to several pathologies such as cancer, Parkinson’s disease, inflammation, diabetes, memory dysfunction, and cardiac hypertrophy [4,5,6]. 

As eukaryotic organisms, fungi also process extracellular signals through MAPK cascades that conserve the architecture described above (Figure 1). These signaling pathways are specialized to face the different conditions that a fungus might encounter, such as high osmolarity concentrations, cell wall aggressions, mating pheromones, and, in certain cases, the presence of host factors or signals that trigger morphological transitions. Understanding the functioning of MAPK cascades in these organisms is particularly important since they are involved in the virulence of several human (e.g. *Candida albicans*, *Cryptococcus neoformans*, and *Aspergillus fumigatus*) and plant pathogens (e.g. *Ustilago maydis*, *Pyricularia oryzae*-sexual morph: *Magnaporthe oryzae-*, and *Ashbya gossypii*) [7,8]. The budding yeast model organism *Saccharomyces cerevisiae* has been the staple in the study of fungal MAPK signaling for its simplicity, easy handling, and genetic tractability. Many of the discoveries from research on the budding yeast have been translated not only to filamentous or dimorphic fungi, but also to higher eukaryotes. In *S. cerevisiae*, four different MAPKs were identified that regulate the high osmolarity response (Hog1), the pheromone response (Fus3), the pseudohyphal and invasive growth upon nutrient deprivation (Kss1), and the cell wall repair and integrity (Slt2). There is a fifth MAPK in *S. cerevisiae,* Smk1, which participates in spore wall formation, but no other elements of the MAPK module have been discovered yet [8,9,10]. In general, the main elements of the mating, high osmolarity (HOG), and cell wall integrity (CWI) MAPK pathways in fungi are conserved. These pathways are mediated by MAPKs Spk1, Sty1 and Pmk1 in the fission yeast *Schizosaccharomyces pombe*, and by Cek1/2, Hog1, and Mkc1 in the dimorphic model yeast *C. albicans* (Figure 1) [11,12]. The few compositional differences of the MAPK pathways between yeast and filamentous fungi were described in previous reviews [13,14].

## 2. General Structure and Essential Motifs of *S. cerevisiae* MKPs

The regulation of the signaling flow is executed on multiple levels of a MAPK cascade. Rapid downregulation of the stimulation generally occurs by receptor desensitization or direct dephosphorylation by phosphatases acting on the MAPKKK, the MAPKK, or predominantly the MAPK itself. Ser/Thr or Tyr phosphatases can dephosphorylate the Thr or Tyr, respectively, at the activation loop to inactivate the MAPK. Despite the general assumption that dephosphorylation of either of these two residues is sufficient for MAPK inactivation, recent evidence suggests that some monophosphorylated MAPKs retain some activity [15,16,17]. However, the main negative regulation is attributed to a particular type of phosphatases belonging to the family of dual specificity phosphatases (DSPs), the MAPK phosphatases (MKPs), which eliminate the phosphate group of both Thr and Tyr residues. MKPs regulate not only the magnitude and duration of MAPK signaling, but also the subcellular localization and substrate selectivity of MAPKs [18]. 

The general structure of MKPs includes a non-catalytic N-terminal domain and a C-terminal catalytic domain that contains a wide pocket with the critical Cys and Arg catalytic residues within the conserved signature HCXXGXXR. An aspartic residue upstream of this signature is also essential for catalysis. Within the N-terminal domain, a MAP kinase interaction motif (KIM), also called docking-domain or D-domain, is characteristic and defined by the presence of a cluster of basic residues followed by a hydrophobic sub-motif containing Leu, Ile, or Val separated by one residue: [K/R](1-3)-X(2–6)-[L/I/V]-X-[L/I/V] [19,20,21]. This domain is also found in other MAPK interactors, such as MAPKKs and MAPK substrates. The positive charge of the D-domain interacts with a negatively charged region at the MAPK called the common docking domain (CD). 

**Figure 2:**
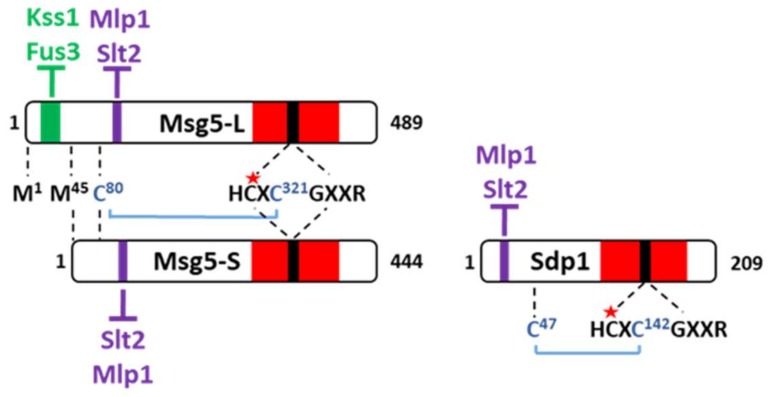
Diagram of the domain composition of *S. cerevisiae* MKPs Msg5, and Sdp1. Msg5-L corresponds to the long translational isoform of Msg5 and Msg5-S to the shorter one. The docking (D)-domain, IYT-motif, and phosphatase domain are drawn in green, purple, and red, respectively. The active site is represented in black and, inside this signature, the essential catalytic cysteine residue is labelled with a red star. The disulfide bond between the non-catalytic cysteine residue within the active site and its upstream cysteine partner is illustrated in blue. The target MAPKs are represented in green or purple, depending on the docking site involved in the binding.

Though mammalian cells contain at least 10 different MKPs, fungal cells only contain one or two. Among the putative or defined DSPs in *S. cerevisiae* (Yvh1, Cdc14, Pps1, Tep1, Msg5, Siw14/Oca3, Oca1, Oca2, Oca4, Oca6, and Sdp1), only Msg5 and Sdp1 have been shown to display MKP activity [22]. These two MKPs are encoded by paralogue genes likely originated from the ancient whole genome duplication that occurred in *S. cerevisiae* [23]. Msg5 is a 489 aa protein that negatively regulates Fus3 [24] and Slt2 [25] and presents the prototypical structure of MKPs, with a regulatory N-terminal domain and a catalytic phosphatase C-terminal domain (Figure 2). As an MKP, the cysteine 319 in its catalytic pocket is essential for its phosphatase activity. Msg5 possesses two different motifs that define its binding with the MAPKs. Msg5 bears a typical D-domain N-terminally located that mediates a canonical interaction with the CD domains of MAPKs Fus3 and Kss1. On the other hand, an unusual motif composed of Ile, Tyr, and Thr (IYT) located at positions 102–104 mediates the interaction with both Slt2 and the Slt2 pseudokinase paralogue Mlp1 through a CD-independent mechanism [26]. 

Notably, Msg5 is not a single species, but is produced as two forms due to alternative translational initiation sites [25]. The short form lacks the first 44 amino acids and therefore does not contain the N-terminal D-domain, implying that full-length Msg5 would be able to act on both mating and CWI MAPKs, whereas the short form only would act on Slt2 (Figure 2). The physiological significance of the existence of these two forms of Msg5 remains to be established but it is tempting to speculate that it could constitute a mechanism for differential regulation of distinct MAPKs by the same MKP. 

Sdp1 is only 209 amino acids long and its very short N-terminal domain presents an IYT motif that mediates interaction with Slt2 and Mlp1, but lacks the Msg5 D-domain counterpart [27]. The absence of this D-domain prevents the interaction of Sdp1 with Fus3 and Kss1, which explains why this MKP acts exclusively on the CWI pathway. The catalytic activity of both Msg5 and Sdp1 resides in the C-terminal part of the protein and, as in all MKPs, a cysteine residue at the active site is essential for its function (Figure 2).

Finally, both Msg5 and Sdp1 display enhanced catalytic activity under oxidative conditions. These phosphatases use an intramolecular disulfide bridge to recognize tyrosine-phosphorylated MAPK substrates. The bridge (Cys47–Cys142 in Sdp1 and Cys80–Cys321 in Msg5) involves a cysteine located two residues downstream of the conserved catalytic cysteine within the active site and an upstream cysteine partner out of the catalytic domain (Figure 2). This disulfide bond is critical for optimal activity of these MKPs and participates in a molecular mechanism that couples oxidative stress with substrate recognition [28]. 

## 3. Structural Conservation of Fungal MKPs

**Figure 3:**
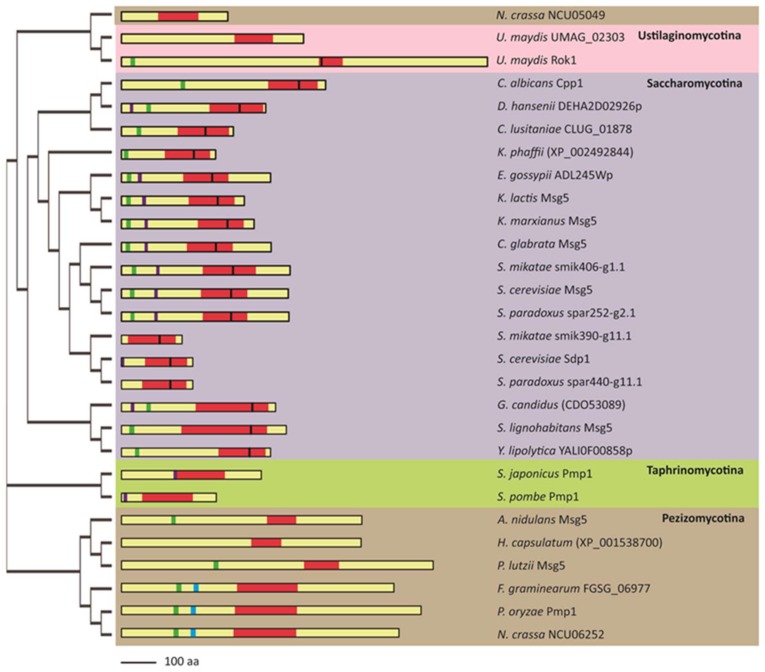
Phylogram and scaled scheme of the domain composition and taxonomic classification of the ScMsg5 orthologues from different fungal species. The phylogram was obtained by multiple protein sequence alignment, using Clustal Omega program (European Bioinformatics Institute (EMBL-EBI), Hinxton, UK) at default settings, of ScMsg5 orthologues selected from National Center for Biotechnology Information (NCBI), Kyoto Encyclopedia of Genes and Genomes (KEGG), or fungal genome databases [*Saccharomyces* Genome Database (SGD), *S. pombe* database (PomBase), *Candida* Genome Database (CGD), and *Aspergillus* Genome Database (AspGD)]. Proteins included are *Neurospora crassa* NCU05049 (XP_956423), *Ustilago maydis* UMAG_02303 (XP_011388628), *Ustilago maydis* UMAG_03701/Rok1 (XP_011390174), *Candida albicans* Cpp1 (XP_723551), *Debaryomyces hansenii* DEHA2D02926p (XP_458594), *Clavispora lusitaniae* CLUG_01878 (XP_002618419), *Komagataella phaffii* (formerly called *Pichia pastoris*) (XP_002492844), *Eremothecium gossypii* (also known as *Ashbya gossypii*) ADL245Wp (NP_983851), *Kluyveromyces lactis* KLLA0_F03597g/Msg5 (XP_455243), *Kluyveromyces marxianus* Msg5 (XP_022678215), *Candida glabrata* CAGL0G01320g/Msg5 (XP_446419), *Saccharomyces mikatae* (smik406-g1.1), *Saccharomyces cerevisiae* Msg5 (NP_014345), *Saccharomyces paradoxus* (spar252-g2.1), *Saccharomyces mikatae* (smik390-g11.1), *Saccharomyces cerevisiae* Sdp1 (NP_012153), *Saccharomyces paradoxus* (spar440-g11.1), *Galactomyces candidus* (CDO53089), *Sugiyamaella lignohabitans* Msg5 (XP_018733395), *Yarrowia lipolytica* YALI0F00858p (XP_504838), *Schizosaccharomyces japonicus* Pmp1 (XP_002174422), *Schizosaccharomyces pombe* Pmp1 (NP_595205), *Aspergillus nidulans* AN4544.2/Msg5 (XP_662148), *Histoplasma capsulatum* (XP_001538700), *Paracoccidioides lutzii* Msg5 (XP_002791021), *Fusarium graminearum* FGSG_06977 (XP_011326656), *Pyricularia oryzae* Pmp1 (XP_003712767), and *Neurospora crassa* NCU06252 (XP_962856). The ScanProsite tool (Swiss Institute of Bioinformatics, Laussanne, Switzerland) was used to search for dual specificity phosphatase catalytic (DSPc) domains (red), IYT/S motifs (purple), and D-domains (green for ScMsg5-like D-domain and blue for PoPmp1-like D-domain). All the sequences contain a DSPc domain in the C-terminal region, including the active site signature motif HCXXGXXR. When a second cysteine that could be involved in a regulatory disulfide bridge is present at the fourth position within the active site, a black line is drawn. Several putative D-domains, characterized by the [K/R](1-3)-X(2–6)-[L/I/V]-X-[L/I/V] signature, were found for each protein (Table S2), but only those that aligned with the described ScMsg5 D-domain or the PoPmp1-like D-domain are shown. Different background colors indicate the subphylum to which the species belong: Pezizomycotina (brown), Ustilaginomycotina (pink), Saccharomycotina (blue), and Taphrinomycotina (green).

As mentioned above regarding MAPKs, orthologues of *S. cerevisiae* Msg5 are found across the fungal kingdom (Figure 3). We conducted a comparative analysis of 61 Msg5 orthologous protein sequences from a wide variety of fungi, selected from the genome databases National Center for Biotechnology Information (NCBI), Kyoto Encyclopedia of Genes and Genomes (KEGG), *Saccharomyces* Genome Database (SGD), *S. pombe* database (PomBase), *Candida* Genome Database (CGD), and *Aspergillus* Genome Database (AspGD) (Table S1). Twenty-eight representative proteins from four subphyla (Saccharomycotina, Pezizomycotina, and Taphrinomycotina from phylum Ascomycota, and Ustilaginomycotina from Basidiomycota) were chosen for a deeper analysis of structural diversity. Although the structure, function, and/or regulation of some of these fungal MKPs are already known, e.g. *S. cerevisiae* Msg5 and Sdp1, *U. maydis* Rok1, *C. albicans* Cpp1, *S. pombe* Pmp1, and *P. oryzae* Pmp1, most of these proteins have not yet been characterized. 

As shown in Figure 3, the phylogram resulting from the multiple protein sequence alignment of these fungal MKPs indicates that proteins from species belonging to the same subphylum cluster together, with the exception of *N. crassa* NCU05049, which is distant from the other Pezizomycotina MKPs. This phylogram reflects the evolutionary branching of fungal MKPs. An important issue to highlight is that only one MKP has been found in most species, except in *S. cerevisiae*, *S. mikatae*, *S. paradoxus*, *U. maydis*, and *N. crassa*, which present two MKPs. In these cases, one of them is similar to ScMsg5 (*S. mikatae* smik406-g1.1, *S. paradoxus* spar252-g2.1, *U. maydis* Rok1, and *N. crassa* NCU06252), whereas the other one is closer to ScSdp1 (*S. mikatae* smik390-g11.1, *S. paradoxus* spar440-g11.1, *U. maydis* UMAG_02303, and *N. crassa* NCU05049) (Figure 3). 

The size of fungal MKPs ranges from 177 (*S. mikatae* smik390-g11.1) to 1069 amino acids (*U. maydis* Rok1). In general, the larger fungal MKPs belong to Pezizomycotina, whereas the smaller ones are present in Saccharomycotina. All of them contain the distinctive active site signature motif HCXXGXXR within the typical dual specificity phosphatase catalytic region (DSPc), which is similar in size except for *U. maydis* Rok1, which displays a notably short DSPc (Figure 3). In agreement with previous observations [28], we found that the regulatory cysteine within the active site implicated in the formation of an intramolecular disulfide bridge required for full activity is conserved in all Saccharomycotina MKPs analyzed, but not in other subphyla excepting *U. maydis* Rok1 (Figure 3). 

The scanning of sequences matching the consensus D-domain yielded several hits within each fungal MKP (Table S2). In the case of ScMsg5, only the one included between amino acids 29 and 38 has been proven to be responsible for its interaction with MAPKs Fus3 and Kss1 [26]. An equivalent D-domain was found in most Msg5-like MKPs of different subphyla, except in Taphrinomycotina (Figure 3), suggesting that a similar interaction mechanism with the corresponding MAPKs could be widely occurring in fungi. However, the presence of the IYT motif, known to mediate the non-canonical binding of ScMsg5 and ScSdp1 to the CWI MAPK Slt2 [27], seems to be restricted to yeast species of Saccharomycotina and Taphrinomycotina. Notably, some yeast MKPs only contain the IYT domain but not the D-domain, namely ScSdp1, SpPmp1, and SjPmp1. This could reflect their specialization in downregulating the CWI pathway. 

Within Saccharomycotina, the only MKPs lacking both interaction domains are *S. mikatae* smik390-g11.1 and *S. paradoxus* spar440-g11.1, even though they contain other hypothetical D-domains (Table S2), suggesting the probable use of alternative motifs for binding to the corresponding MAPKs. 

*P. oryzae* Pmp1 has been shown to directly downregulate CWI MAPK Mps1. The region responsible for the interaction with Mps1 is highly conserved in other filamentous fungi [29] (Figure 3). This region does not include the ScMsg5-like D-domain, but another putative D-domain (Table S2) that could mediate the interaction with MAPK. 

In conclusion, this analysis evidences the wide variety of interaction strategies and regulatory mechanisms that fungal MKPs possess and suggests several points to be further explored and extended to the putative fungal MKPs not yet characterized. 

## 4. Function of Fungal MKPs

### 4.1. Msg5 Regulates the S. cerevisiae Mating Pathway by Targeting Fus3 

*MSG5* was initially identified as a multicopy suppressor gene of the cell cycle arrest of haploid *gpa1* mutants [24], which bear a mutation that leads to the constitutive activation of the mating pathway. *MSG5* overexpression causes cells to overcome pheromone-induced arrest and reduces the expression of a typical transcriptional readout of the mating pathway. In this seminal work, Msg5 was also proven to act as a phosphatase that was able to inactivate Fus3 in vitro (Figure 1a). Since *MSG5* was observed to be transcriptionally induced in response to pheromone exposure and the loss of this phosphatase increased the sensitivity of cells to pheromones, Msg5 was proposed to promote adaptation to this stimulus [24]. A negative effect on the mating pathway was soon defined for the protein tyrosine phosphatases Ptp2 and Ptp3, which dephosphorylate the same target, the MAPK Fus3. However, whereas Ptp2 and Ptp3 are critical in maintaining the mating pathway at a low basal activity in the absence of pheromone, Msg5 primarily participates in the inactivation of Fus3 following stimulation [30]. By employing both mathematical models and experimental data, the feedback inhibition of Fus3 by Msg5 has been proposed to be the most significant in the mid-to-late time points after pheromone-induced activation of this pathway. In contrast, the feedback inhibition of Fus3 by Ptp3 is most impactful in the early time points after the pheromone exposure [31]. 

Msg5 also seems to promote adaptation by regulating the subcellular distribution of Fus3. Pheromone recovery is correlated with a decrease in nuclear Fus3 [32] and, although Msg5 is distributed evenly in both the nucleus and the cytoplasm in a pheromone-independent manner, it counteracts the nuclear accumulation of Fus3 by dephosphorylating this MAPK in both cellular compartments [33]. The mechanisms underlying the downregulation of Fus3 by Msg5 also includes the involvement of monophosphorylated forms of the MAPK. A substantial pool of Fus3, which is only phosphorylated in Tyr182—the conserved Tyr residue of the MAPK activation motif—was shown to be generated in pheromone-stimulated cells. This pool of Tyr-phosphorylated kinase shows an inhibitory effect on signaling and acts in opposition to the fully phosphorylated Fus3. Importantly, cells lacking Msg5 not only show an increase in dual-phosphorylated Fus3, but also a decrease in monophosphorylated Fus3, indicating that Msg5 is involved in maintaining a pool of this inhibitory MAPK form [16]. 

Although overexpression of Msg5 reduces the amount of phosphorylated Kss1, this MAPK does not seem to be directly regulated by the MKP [34]. Removal of Msg5 does not result in increased Kss1 phosphorylation but in decreased Kss1-driven FRE(Tec1)-*lacZ* expression, probably due to a Fus3-mediated effect [35]. 

### 4.2. CWI MAPK Slt2 is Regulated by the MKPs Msg5 and Sdp1 in S. cerevisiae

The ability of Msg5 to suppress the lethality promoted by the overexpression of *MKK1P396*, coding a hyperactive version of the MAPKK Mkk1, suggests the possibility that this DSP also acts on the CWI pathway [36]. It was later clarified that Msg5 downregulates this route since overexpression of *MSG5* eliminates the high Slt2 phosphorylation of cells lacking the Rho1 GAP Sac7, whereas disruption of *MSG5* in wild-type cells resulted in increased phospho-Slt2 levels, both at basal conditions and under cell wall stress [37]. When Msg5 was shown to bind and dephosphorylate Slt2 in vitro, this MAPK was formally identified as the target of Msg5 within the CWI pathway [25] (Figure 1a). 

As occurs with other negative regulators, *msg5*∆ cells display sensitivity to cell wall stress [25], a phenotype that is likely derived from an increased CWI signaling, similar to that occurring in cells lacking the serine/threonine phosphatase Ptc1 [38]. This is also likely the mechanism underlying the vacuolar fragmentation observed in double *ptp2 msg5* mutants [39], a phenotype displayed by *ptc1* mutants and dependent on an exacerbated Slt2-mediated signaling [38]. However, although Msg5 is involved in the activation of Slt2 in the response to genotoxic stress, *msg5* mutant cells do not show sensitivity but tolerance to this stress. This could be explained by the fact that, under this particular insult, the signal enters the CWI pathway at the Slt2 level due to the degradation of Msg5 (see below), without the participation of upstream components and likely involved different outputs [40]. 

In contrast to Msg5, Sdp1 does not seem to regulate Slt2 in basal conditions. This phosphatase has been shown to act only under heat stress [41] and to be required for efficient Slt2 dephosphorylation during heat shock adaptation [42]. Whereas Sdp1 localizes both in nucleus and cytoplasm in basal conditions, it becomes localized in punctate spots throughout the cells upon heat shock. Many of these puncta colocalize with mitochondria, and Sdp1 reappears in the nucleus after prolonged heat stress, concurrent with Slt2 dephosphorylation [41]. 

### 4.3. C. albicans MKP Cpp1 Participates in Phenotypic Transition, Mating, and Virulence

Further knowledge about the functional relevance of MKPs within the subphylum Saccharomycotina was provided by studies on the dimorphic fungus and opportunistic pathogen *C. albicans.* The importance of fungal MKPs in morphogenesis is also illustrated in the case of *C. albicans* Cpp1 protein. This MKP is a negative regulator of filamentous growth and its removal derepresses hyphal formation under non-inducing conditions. This phenotype is eliminated if the MAPK Cek1 (the ScKss1 orthologue) is removed [43]. As expected, soon after this work, the Cek1 MAPK pathway was shown to be negatively regulated by Cpp1 [44] (Figure 1c). Surprisingly, Cpp1 represses hyphal gene expression by acting through a Cek1p-independent mechanism [45]. Cpp1 has been proposed to act as a key element in the crosstalk between MAPKs Hog1 and Cek1, since *hog1* and *cpp1* deletion mutants share hyperfilamentation and opaque cell formation phenotypes and display increased Cek1 activation. Moreover, *CPP1* expression is positively regulated by Hog1 [46]. 

Loss of Cpp1 was reported to activate the pheromone response pathway in a Cek1-dependent manner, indicating an additional role of this MKP in the mating pathway. These authors also found that basal Cek2 phosphorylation is increased in *cpp1* mutants, suggesting that Cpp1 could be downregulating Cek2 as well (Figure 1c) [47]. 

Pathogenicity of a homozygotic *cpp1* mutant strain is reduced in murine models of candidiasis, clarifying that Cpp1 is a virulence determinant [43]. Accordingly, it is transcriptionally induced within the human host during thrush [48]. The role of Cpp1 as a virulence factor has been connected with its activity on the target MAPK. The constitutive activation of Cek1 by deletion of its phosphatase Cpp1 increases susceptibility of cells to the protein Hst5, a salivary fungicidal histidine-rich protein constitutively produced by human salivary gland cells. Cek1 activation likely enhances Hst5-mediated killing in part through exposure of cell wall β-1,3-glucans [49].

### 4.4. S. pombe Pmp1: The Only Characterized MKP Within the Subphylum Taphrinomycotina

The MKP Pmp1 is the main phosphatase acting on the fission yeast MAPK Pmk1, which is orthologous to the MAPK Slt2 [50]. Pmp1 is a cytoplasmic protein, suggesting that Pmk1 inactivation by this phosphatase most likely occurs in the cytoplasm [51]. Removal of Pmp1 promotes an increase in signaling through the mating pathway [52], although the MAPK Spk1 has not been formally proven to be regulated by this MKP. 

Pmp1 plays an important role in Cl^−^ homeostasis. It was originally identified as a multicopy suppressor of the chloride hypersensitivity of a calcineurin mutant, since calcineurin and Pmk1 MAPK pathways play antagonistic roles in the regulation of the Cl^−^ homeostasis [50]. Thus, cells lacking Pmp1 display a strong sensitivity to this anion [50]. Pmp1 also plays a critical role in morphogenesis. Mutant cells lacking this MKP present an aberrant morphology, frequently round or pear-shaped cells [50], and multiseptated and defective cell separation phenotypes [53]. Tight regulation of Pmk1 activity through Pmp1 is thus important in *S. pombe* cytokinesis (Figure 1b). 

### 4.5. Filamentous Fungi MKPs are Involved in Mycelial Growth and Virulence 

MKPs have been identified in other fungi, including some belonging to the subphylum Pezizomycotina, such as *Neurospora crassa*, *Fusarium oxysporum*, *Madurella mycetomatis*, *Gaeumannomyces graminis*, *Verticillium dahliae*, *Colletotrichum graminicola*, and *Ustilaginoidea virens* [29] (Table S1). However, only functional characterization of Msg5 homologues from filamentous fungi *F. graminearum* and *P. oryzae* has been completed to date. 

In the wheat scab fungus *F. graminearum*, the Mgv1 MAPK pathway is homologous to the cell wall integrity (CWI) pathway in budding yeast and is important for plant infection [54]. The Msg5 orthologue, FgMsg5, was shown to dephosphorylate Mgv1 [55], and its elimination led to mycelial growth defects [56]. 

A functional study with the rice blast fungus *P. oryzae* Pmp1 not only identified this MKP as a negative regulator of the MAPK Pmk1 (Fus3 orthologue) but also of the CWI MAPK Mps1 (Slt2 orthologue), both essential for pathogenesis. In contrast to that observed for Mps1, yeast two-hybrid analysis revealed the absence of direct interaction of Pmp1 with Pmk1, opening up the possibility of the existence of some adaptor between both proteins. ∆*pmp1* mutants presented a reduced mycelial growth and conidiation and, more importantly, reduced virulence in both rice and barley [29]. However, further research is needed to provide more information about additional substrates of Pmp1, since both ∆*pmk1* and ∆*mps1* mutants develop a normal mycelial growth [57,58]. 

### 4.6. Choose the Right Host: A Role for MKPs Within Basidiomycota

MKP activity has also been proven to play a role in morphogenetic programs and disease development in Basidiomycota. This is the case of the causative agent of corn smut, *Ustilago maydis*. In this fungus, the partially redundant MAPKs Kpp2 and Kpp6 are regulated by the MKP Rok1. The lack of Rok1 activity promotes increased filamentation and enhanced virulence, whereas overexpression of *rok1* induces the opposite effect. The reason for this fungus not losing Rok1 through evolution could be that *rok1* mutants can no longer differentiate whether they are on the correct plant host [59]. Albeit the apparent dispensability of this fungal MKP in phenotypic assays, the precise control of MAPK phosphorylation is likely an advantage for *U. maydis* cells in nature.

## 5. Regulatory Mechanisms of Fungal MKPs

**Figure 4:**
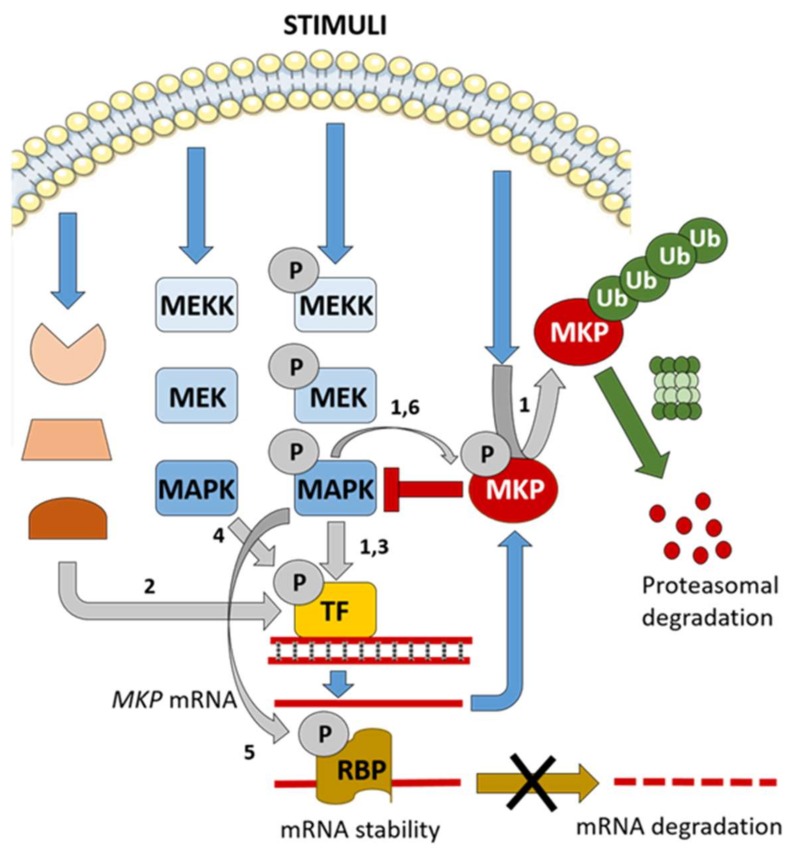
Multiple levels of MKP regulation in fungi. In response to stress, MKP gene expression can be transcriptionally induced by a transcription factor (TF) activated by either the MAPK target of the MKP, another related MAPK pathway, or another route. The stability of the MKP mRNA can be post-transcriptionally regulated by an RNA-binding protein (RBP), which increases the mRNA half-life and thereby the amount of MKP protein. The activity of the RBP can be in turn modulated by phosphorylation by the target MAPK. After translation, the MKP can be further regulated by different post-translational modifications, such as phosphorylation or ubiquitination. MKP phosphorylation is usually exerted by its target MAPK and ubiquitination generally leads to MKP degradation by the proteasome. Numbers designate the fungal MKPs known to be under the regulatory mechanisms indicated by grey arrows: (**1**) *S. cerevisiae* Msg5, (**2**) *S. cerevisiae* Sdp1, (**3**) *U. maydis* Rok1, (**4**) *C. albicans* Cpp1, (**5**) *S. pombe* Pmp1, and (**6**) *P. oryzae* Pmp1.

Like in mammalian MKPs, different regulatory mechanisms operating on fungal MPKs provide distinct layers of control on MAPK pathways (Figure 4). One of these mechanisms relies on the MAPK-dependent transcriptional induction of MKPs under stimulating conditions. This generates a negative feedback loop that maintains an adequate MAPK activation level and/or promotes adaptation [60]. In *S. cerevisiae*, transcription of *MSG5* is regulated by both Msg5-regulated pathways: mating and CWI [24,61]. Whereas Msg5 is primarily involved in adaptation to pheromone exposure in the mating pathway, its main role in the CWI pathway is the control of the maximum intensity of signaling flow through the pathway [22]. *U. maydis* Rok1 is induced in response to the activation of its specific MAPK pathway [59]. In contrast, the *S. cerevisiae* Slt2-selective MKP Sdp1 is transcriptionally induced by heat stress, but in a CWI-independent manner. This induction is partially mediated by the transcription factors Msn2/4 of the cAMP-PKA pathway [41], which suggests a linkage between these two pathways through Sdp1. Therefore, the transcriptional regulation of MKPs can be exerted not only by their substrate MAPKs but also by other signaling pathways not targeted by the MKP, allowing signaling cross-inhibition. This is also the case for Cpp1, the Cek1-specific MKP from *C. albicans* that has been reported to mediate the crosstalk between Hog1 and Cek1 MAPK pathways involved in both yeast-hyphae transition and white-opaque switching [46]. In that work, Hog1 was shown to downregulate Cek1 signaling by inducing the expression of Cpp1. 

The increase in mRNA levels can be achieved not only by transcriptional upregulation but also by post-transcriptional mechanisms, including control of mRNA stability [62]. Such regulation has been reported for Pmp1, the MKP of the CWI MAPK Pmk1 in *S. pombe*. The RNA-binding protein (RBP) Rnc1 was shown to bind and stabilize an otherwise unstable Pmp1 mRNA [63]. A similar RBP-mediated post-transcriptional mRNA stabilization mechanism has also been reported for mammalian MKPs, such as MKP-1 [64]. *S. pombe* Rnc1 is phosphorylated at Thr^50^ by the MAPK Pmk1, mediating a novel type of negative-feedback regulatory loop in MAPK signaling pathways. This phosphorylation positively regulates the RNA-binding activity of Rnc1, resulting in stabilization of the Pmp1 mRNA and the subsequent increase in Pmk1 dephosphorylation [63]. 

Transcriptional and post-transcriptional regulation is often combined with post-translational mechanisms to ensure tight control of MKPs. Specific protein modifications, such as phosphorylation or ubiquitination, can alter the subcellular localization, stability, affinity, or catalytic activity of MKPs, resulting in either a reinforcement or a weakening of their action on MAPK signaling [60]. Phosphorylation of fungal MPKs was first described in *S. cerevisiae* Msg5, which is modified by its target MAPKs, Fus3 and Slt2. Phosphorylation of Msg5 by Fus3 was observed in vitro but its occurrence and functional significance in vivo remain to be established [24]. Slt2 was shown to phosphorylate Msg5 in yeast cells under cell wall stress conditions, alongside a decreased interaction between both proteins [25]. These results point to the existence of a positive feedback regulatory loop to reduce the activity of the phosphatase on the MAPK, allowing maximum activation of the CWI pathway. Subsequent *in vitro* kinase assays showed that both the N-terminal regulatory and the C-terminal catalytic domains of Msg5 are susceptible to phosphorylation by Slt2 [65]. However, the elimination of all the Ser/Thr-Pro putative MAPK target sites in Msg5 did not seem to alter its ability to dephosphorylate Slt2 in vivo, suggesting that this regulatory mechanism must be more subtle or complex than expected [65]. Regulatory phosphorylation has been revealed in the *P. oryzae* MKP Pmp1 by phosphoproteomic analysis [29]. Pmp1 was found phosphorylated at Ser^240^, a position that is conserved only in filamentous fungi and lies just downstream of its region of interaction with the CWI MAPK Mps1. Pmp1 phosphorylation at this site was shown to be important for regulating MAPK phosphorylation of both Pmk1 (Fus3 orthologue) and Mps1 (Slt2 orthologue), but the involvement of such regulation in fungal virulence is not yet clear. The kinase responsible for Pmp1 phosphorylation is still unknown, but, curiously, Ser^240^ does not exactly match a canonical Ser/Thr-Pro MAPK target motif, although it is immediately followed by a Ser-Pro sequence [29]. 

Ubiquitination is another post-translational regulatory mechanism that allows cells to modulate signaling. Although ubiquitination can affect proteins in different ways, this modification usually results in the proteasomal degradation of the protein [60]. MAPKs can trigger proteolysis of their specific MKPs via the ubiquitin-proteasome pathway to enable long-term activation. This positive feedback loop is operating, for example, in the mammalian ERK1/2 pathway. MKP-1 stability has been reported to be regulated by ERK-mediated phosphorylation, which facilitates subsequent ubiquitination and degradation [66]. In *S. cerevisiae*, ubiquitin-mediated proteolysis of Msg5 has been proposed as the mechanism responsible for Slt2 activation in response to genotoxic stress [40]. In contrast to cell wall stress, which involves the stimulation of the whole CWI pathway, the phosphorylation of Slt2 by DNA damage does not require the activation of upstream protein kinases of the MAPK module. The degradation of Msg5 triggered by genotoxic stress depends on the presence of Slt2 but is independent of its kinase activity. Therefore, this mechanism does not seem to fit with the classical MAPK-mediated positive feedback loop described above. Slt2 activation by DNA damage does not lead to the general transcriptional pattern associated with the CWI pathway, suggesting the existence of unknown genotoxic stress-specific targets. 

In summary, fungi display a similar complexity in the regulation of MPKs as other eukaryotic organisms. The regulation of MPKs can be exerted at multiple steps: Transcriptional, post-transcriptional, and post-translational, allowing a fine-tuning multifaceted modulation of MPK levels, thereby determining the duration and strength of MAPK signaling. 

## 6. Concluding Remarks

So far, most studies on fungal MKPs focused on model yeast species, namely, *S. cerevisiae*, *S. pombe*, and *C. albicans*. However, orthologues of MKPs are found in all fungal proteomes examined, indicating broad conservation of this type of protein phosphatases in both pathogenic and environmental fungi. Although MKPs are very important in fungal biology as key negative regulators of MAPKs, most of them are yet uncharacterized. Hence their study will be crucial in future research. MKPs exert wide and complex functions on cellular signaling due to their ability not only to modulate but also to be regulated by several MAPK pathways. Therefore, they play an essential role in coordinating different routes and providing response plasticity to diverse environmental stimuli. 

Fungal MKPs share structural and regulatory features with mammalian MKPs, such as typical docking and catalytic domains, and common transcriptional, post-transcriptional, and post-translational mechanisms that control MKP protein abundance and activity. Due to this conservation across evolution, knowledge generated in model yeasts is very helpful to mammalian MKPs researchers and vice versa. For example, the finding in yeast of a mechanism of MAPK activation through degradation of its MKP, triggered by a specific stress that does not stimulate the MAPK cascade, could inspire the search for similar cases in higher eukaryotic organisms. Nevertheless, fungal MKPs also display unique characteristics, such as disulfide bridge-mediated catalytic activation and distinctive MAPK interaction domains, which are even specific to particular fungal subphyla. These peculiarities likely reflect the different environments that fungal species have faced throughout evolution. Involvement of MKPs in fungal virulence leads us to speculate that differences between fungi and mammalian MKPs might be exploited for developing antifungal drugs with high selective toxicity.

## Supplementary Materials

Supplementary Materials can be found at https://www.mdpi.com/1422-0067/20/7/1709/s1.

## Abbreviations

MAPK	Mitogen Activated Protein Kinase
MKP	MAP Kinase phosphatase
DUSP	Dual Specificity Phosphatase
